# A diagnostic conundrum in Bardet–Biedl syndrome: when genetic diagnosis precedes clinical diagnosis

**DOI:** 10.1530/EDM-23-0055

**Published:** 2023-11-24

**Authors:** Nele Van Roy, Sylvester Heerwegh, Dashty Husein, Joke Ruys, Peter Coremans

**Affiliations:** 1 Department of Diabetes and Endocrinology, Vitaz, Sint-Niklaas, Belgium; 2 Department of Ophthalmology, Vitaz, Sint-Niklaas, Belgium

**Keywords:** Adolescent/young adult, Female, Other, Belgium, Hypothalamus, Genetics and mutation, Paediatric endocrinology, Ophthalmology, Error in diagnosis/pitfalls and caveats, November, 2023

## Abstract

**Summary:**

Bardet–Biedl syndrome (BBS) is a rare, autosomal recessive, multisystem non-motile ciliopathy of progressive onset. It is primarily characterised by rod–cone dystrophy, early-onset obesity and related complications, postaxial polydactyly, renal and genitourinary abnormalities, learning disabilities, and hypogonadism. The diagnosis is based on Beales’ modified diagnostic criteria. We present a case of two monozygotic female twins, 17 years of age at presentation, referred for obesity since childhood. The initial hormonal work-up was negative and no dysmorphic features were noted. They were diagnosed with exogenous obesity. However, after ophthalmologic problems became apparent, rod–cone dystrophy was observed and genetic testing was performed. A mutation in the BBS2 gene led to the diagnosis of BBS, although the full diagnostic criteria were not met. This case not only highlights the need to raise awareness for BBS but also exposes two limitations of the current diagnostic standard. The first limitation is the low sensitivity of the clinical diagnostic model, due to the progressive onset and the high variability of the syndrome. The second limitation is the unclear role of genetic testing. As genetic testing becomes more widely available, genetic diagnosis preceding clinical diagnosis will become more common, leading to a diagnostic conundrum. We propose an update of the diagnostic model. A less strict application in the presence of confirmed genetic mutations should be applied, as this could facilitate earlier diagnosis and intervention. This is important because therapeutic agents are being developed that could have a significant impact on quality of life and prognosis.

**Learning points:**

- Due to the low prevalence, the significant inter-and intrafamilial variation, and the slowly evolving phenotype, monogenic forms of obesity such as Bardet–Biedl syndrome are difficult to diagnose. Despite advances in the understanding of the presentation, pathophysiology and access to accurate genetic characterisation, a substantial number of diagnoses are still made by ophthalmology, as recognition of BBS in other departments of medicine, remains limited.
- Clinical diagnosis of BBS is based on Beales’ modified diagnostic criteria which require the presence of four primary features or three primary features plus two secondary features. This model has its limitations. Due to the progressive onset of clinical symptoms, patients generally do not meet the diagnostic criteria early in life, leading to a delay in diagnosis. In addition, the role of genetic testing remains controversial. However, as it becomes more widely available, genetic diagnosis may precede a full clinical diagnosis.
- BBS has an impact on the quality of life and prognosis of both the patient and the family. Obesity management strategies are an important part of the multidisciplinary approach, as there is no cure available. Setmelanotide has shown promising results in a phase 3 trial, but its effect in clinical practice remains unproven.

## Background

Obesity is a heterogeneous disorder, mostly the result of a complex interaction between polygenic predisposition and environmental factors. Less than 5% of adult obesity can be explained by genetic alterations in single genes: monogenic obesity (1). BBS is an autosomal recessive, monogenic syndrome of obesity. The prevalence is estimated to be 1:160,000 in Northern Europe but is subject to geographical variation and is influenced by consanguinity. BBS is caused by mutations in one of the currently described 26 genes that play an important role in the function of primary cilia (2). Abnormalities in the ciliary function result in a variety of symptoms, some of which with invalidating character. Early-onset obesity, postaxial polydactyly, rod–cone dystrophy, renal or genitourinary abnormalities, learning disabilities, and hypogonadism are considered primary features. Diabetes, speech deficit, hearing loss, anosmia, cardiomyopathy, and hepatic fibrosis are described as secondary features. Diagnosis is based on Beales’ modified diagnostic criteria: the presence of four primary features or three primary plus two secondary features is required (3). Although genetic testing is becoming more widely available, its role remains unclear. The phenotype evolves slowly during the first decades of life, leading to a significant variation in clinical presentation and resulting in a mean age of diagnosis of 9 years (2, 3). The prevalence of metabolic syndrome is significantly higher in patients with BBS. Primary renal failure and secondary cardiovascular disease are the major causes of morbidity and early mortality. The prognosis is therefore curbed, with a median survival of 63 years (4, 5). To date, there is no cure for BBS. Therefore, early diagnosis remains important as treatment is limited to aggressive management of comorbidities and early educational planning to reduce the impact of vision loss (2, 6).

Here we present a case of two monozygotic twins with late presentation of early-onset obesity. Although they did not meet the full clinical diagnostic criteria for BBS, a diagnosis of BBS was made with the aid of genetic testing.

## Case presentation

Two 17-year-old female monozygotic twins of Palestinian origin were referred to the endocrinology department for class 1 obesity. They were born in a consanguineous marriage and had seven siblings (ages 3–20). Both had normal birth weight but showed rapid weight gain during childhood. Food-seeking behaviour was not documented. Exercise was limited to physical education in school. The age of menarche was 13, and menses were regular. Issue A had a history of strabismus surgery and removal of two supernumerary teeth. Issue B presented to the emergency department on three separate occasions for lower abdominal pain, due to cystitis (first presentation) and urinary retention (second and third presentation). Ultrasound had shown a globus vesicalis with dilatation of the pyelocaliceal system but no other renal or abdominal abnormalities.

On initial contact, the patients were found to have class 1 obesity, a substantially increased waist circumference, stage 1 hypertension, acanthosis nigricans, pale striae, and a buffalo hump. Dysmorphic features were not observed (Table 1).

**Table 1:** Clinical features at first presentation.

	**Issue A**	**Issue B**
Blood pressure, mm Hg	129/87	133/85
Height, cm	156	157.3
Weight, kg	77.4	80.3
BMI, kg/m²	31.8	32.45
Waist circumference, cm	105	97
Acanthosis nigricans	+	+
Striae	+	+
Buffalo hump	+	+
Hirsutism	−	−

## Investigation

Hormonal and metabolic screening was performed (Table 2). Both patients were insulin-resistant, had dyslipidaemia and abnormal liver function tests. Thyroid function tests were within normal limits. Normal suppression of morning cortisol after a low-dose dexamethasone suppression test was seen in issue A; this was not performed in issue B. Based on these results, obesity was considered exogenous, and conservative treatment was initiated.

**Table 2:** Laboratory results.

	**Issue A**	**Issue B**	**Reference values**
Glucose, mg/dL	89	86	70–110
HbA1c, %	6.3	5.4	1.3–5.8
Insulin, pmol/L	126	164	0–174
C-peptide, nmol/L	0.60		0.27–1.28
TSH, mU/L	1.4	0.78	0.4–4.17
T4, pmol/L	11.4	12.5	10.7–18.4
LH, U/L	9.7		
FSH, U/L	5.28		
Oestradiol, ng/dL	112.8		
Testosterone, ng/dL	28.3		11.78–43.34
Free testosterone, ng/dL	0.68		0.06–2.57
SHBG, nmol/L	18.6		19.36–161.78
Cortisol, µg/dL	10.6		6–30
ACTH, µg/dL	7.3		20–88
Cortisol, µg/dL^a^	0.6		6–30
ACTH, µg/dL^a^	<5		20–88
Creatinine, mg/dL	0.69	0.59	0.55–1.02
eGFR (MDRD), mL/min/1.73 m²	>60	>60	>60
GOT, U/L	45	43	15–35
GPT, U/L	62	61	7–35
AP, U/L	66	65	82–317
GGT, U/L	44	46	<38
Cholesterol, mg/dL	217	249	0–200
HDL cholesterol, mg/dL	38	56	>40
LDL cholesterol, mg/dL	145	174	<115
Triglycerides, mg/dL	168	95	<150

^a^1 mg dexamethasone suppression test.

**Figure 1:**
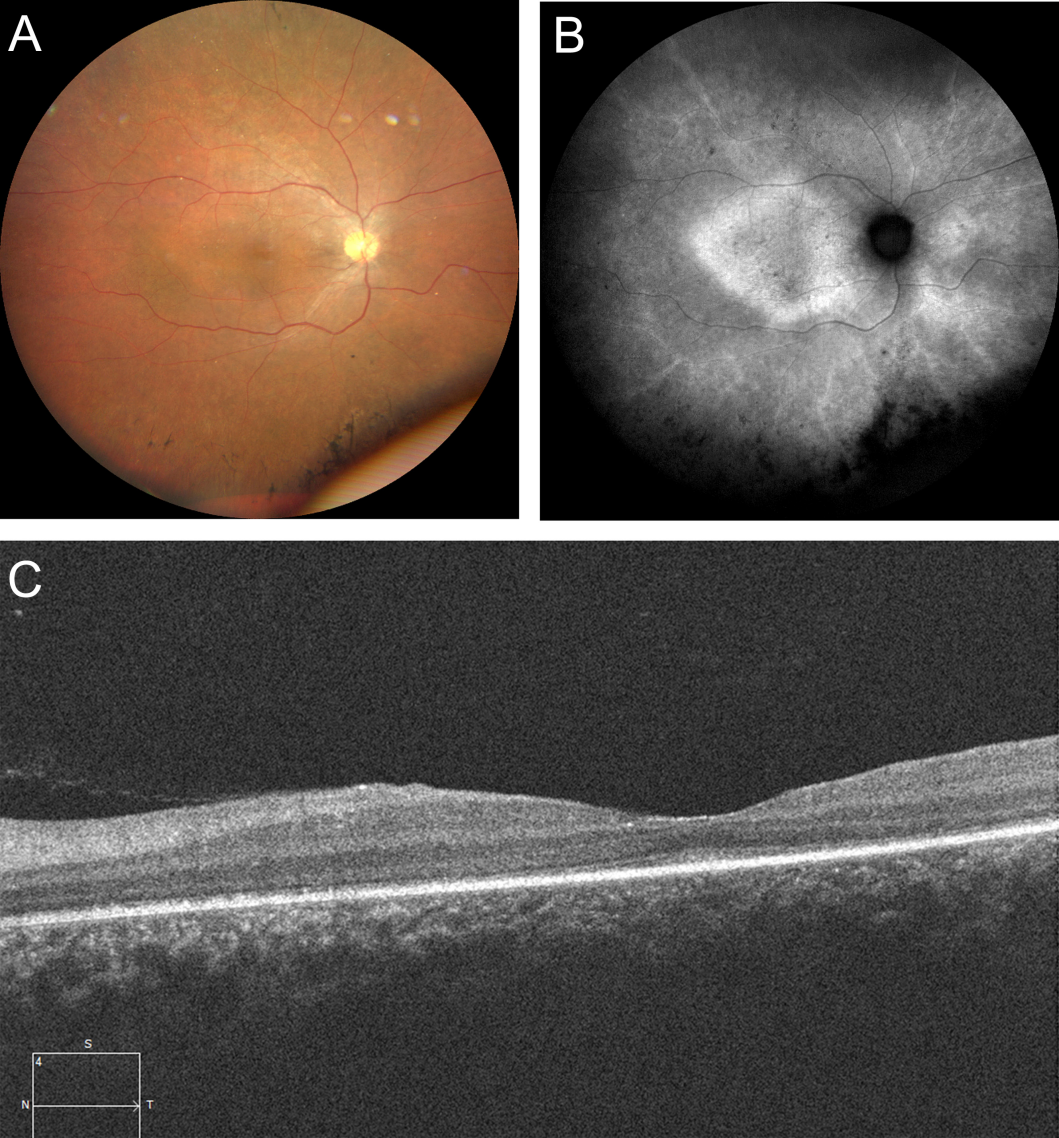
(A) Images of the ocular findings in issue A. Fundoscopy of the right eye: a wavy optic disc, optic nerve pallor, attenuation of retinal blood vessels, and peripheral pigmentary changes. (B) Autofluorescence of the right eye: a hyperautofluorescent ring at the posterior pole, areas of hyperautofluorescence in the periphery, especially surrounding the vessels, and scattered hypoautofluorescent dots. (C) Optical coherence tomography scan: a thinning of the outer retina and disintegration of the ellipsoid zone (EZ) and the external limiting membrane (ELM).

Although visual problems were not mentioned during the first contact, issue A consulted the ophthalmology department 6 months later because of progressive deterioration of sight and photophobia. Ophthalmic examination revealed almost complete loss of visual acuity bilaterally, with loss of peripheral vision. Fundoscopy, autofluorescence imaging and foveal optical coherence tomography scans were consistent with retinitis pigmentosa, a form of rod–cone dystrophy (Fig. 1). An exome-based RetNet panel of 290 genes, designed to screen for inherited retinal diseases, was analysed. A homozygous missense variant in exon 2/17 of the BBS2 gene on chromosome 16 (c.224T>G (p.Val75Gly)) was found, indicating BBS (classified as likely pathogenic).

After genetic testing, the patients were re-evaluated in the endocrinology department for the clinical diagnostic criteria of BBS (Table 3). Although the full clinical criteria were not met, given the results of genetic testing a diagnosis of BBS was confirmed.

**Table 3:** Beales’ modified diagnostic criteria (3), applied to Issue A, Issue B, Family 1 (8) and Family 2 (9), described in the literature.^a^

	**Issue A**	**Issue B**	**Family 1**	**Family 2**
**Primary features**				
Retinitis pigmentosa	+	+	+	+
Polydactyly	−	−	+	°
Obesity	+	+	+	°
Learning disabilities	−	−	+	°
Hypogonadism	−	−	°	°
Renal abnormalities	−	+	°	+
**Secondary features**				
Speech disorder/delay	+	−	°	°
Strabismus/cataract/astigmatism	+	−	°	°
Brachydactyly/syndactyly	−	−	°	°
Developmental delay	−	−	°	°
Polyuria/polydipsia	−	−	°	°
Ataxia/poor coordination/imbalance	−	−	°	°
Mild spasticity	−	−	°	°
Diabetes mellitus	−	−	°	°
Dental crowding/hypodontia/small roots/high arched palate	+	+	°	°
Left ventricular hypertrophy/congenital heart disease	−	−	°	°
Hepatic fibrosis	−	−	°	°

^a^When a characteristic was present in more than half of the family members, it was considered as positive; °Information could not be retrieved from the original article.

## Treatment

Following the initial presentation, a conservative diet- and exercise-based program was initiated. Six months later, after diagnosis of BBS, a trial with dulaglutide, a subcutaneous GLP-1 receptor agonist at a maximum dose of 1.5 mg weekly, was initiated based on previously reported good results in a patient with BBS (7). Despite good compliance, no weight loss was observed. After a 3-month trial, therapy was discontinued.

## Outcome and follow-up

Annual visits to the endocrinology and ophthalmology departments were scheduled. The patients were motivated to maintain a low-calorie diet and exercise under professional guidance. Genetic counselling in the parents and the 7 siblings was provided. Both parents appeared to be carriers of the mutation, as well as two other siblings. None were affected.

## Discussion

We described a case of two 17-year-old monozygotic twins with a late diagnosis of BBS, who initially presented with early-onset obesity. In clinical practice, the diagnosis is based on Beales’ modified diagnostic criteria: the presence of four primary features or three primary and two secondary features is required (3). In our case the patients did not meet the full diagnostic criteria. However, aided by genetic testing, the diagnosis of BBS was confirmed. This case highlights two limitations of the diagnostic model of BBS: its low sensitivity especially at a young age and the unclear role of genetic testing. In addition, this case leads to a reclassification of the identified mutation: an upgrade from likely pathogenic to pathogenic, leading to earlier diagnosis and thus intervention.

The first limitation is inherently due to the high variability and progressive onset of the phenotype, leading to low sensitivity of the diagnostic criteria at young age, and a significant delay in diagnosis. Although this was described for all primary features in the literature, in this case it was most evident for obesity and rod–cone dystrophy (2). The weight pattern associated with BBS is typically characterised by a normal birth weight, followed by rapid weight gain in early childhood due to food-seeking behaviour. However, a highly variable phenotype has been described: overweight (BMI >25 kg/m²) at one point in time is present in 72–92% of cases, class 3 obesity (BMI >40 kg/m²) in 25%. In addition, successful weight loss strategies have been implemented previously, implying that weight is not fixed throughout life (2, 3, 4, 6, 7). Although both patients had early-onset obesity, the typical food-seeking behaviour was not described and they were only referred for endocrinological work-up at the age of 17. Rod–cone dystrophy is an almost universal feature, eventually leading to complete blindness. The mean age at which parents reported night blindness for the first time in their children was 8.5 years, with a range of 1–34 years (2, 3, 4). In this case, ophthalmologic problems only became clear at the age of 18. The observation of rod–cone dystrophy in these patients was the clue for genetic testing.

After the genetic diagnosis of BBS, the patients were re-evaluated. Although additional primary and secondary features were withheld, a strict clinical diagnosis could not be made, highlighting the second limitation to Beales’ diagnostic model: the unclear role of genetic testing. Recent publications suggest that genetic testing can be used to confirm the clinical diagnosis (2, 5). In this case, however, the genetic diagnosis of BBS preceded clinical diagnosis, leading to a diagnostic conundrum.

To date, the identified mutation is considered as likely pathogenic. The identical homozygous single-nucleotide variant c.224T>G (p.Val75Gly) in exon 2/17 of the BBS2 gene on chromosome 16 has been described in four families with confirmed clinical diagnosis of BBS on ClinVar (https://www.ncbi.nlm.nih.gov/clinvar/variation/4569/?oq=BBS2%5bgene%5d+AND+Val75Gly%5bvarname%5d+&m=NM_031885.5(BBS2):c.224T%3EG%20(p.Val75Gly; Accessed on: 24 April 2023). Two of them have been described in the literature (8, 9); patient characteristics are summarised in Table 3. Diagnosis of BBS was confirmed in our patients, although they did not meet the full clinical criteria, given the results of genetic testing. This confirmation implies a fifth family identified, suggesting an upgrade in classification to ‘pathogenic’. Both the confirmation of the diagnosis and the upgrading of the classification lead to an earlier diagnosis and intervention. Although gene therapy is likely to emerge in the future, to date there is no cure for BBS. Early obesity management strategies, including GLP-1 agents, and bariatric surgery have an important role in the multidisciplinary approach to improve both quality of life and prognosis for the patient and their family. Targeted therapy with setmelanotide has shown promising results in a phase 3 trial; however, its effectiveness in clinical practice remains unproven (2, 6).

In conclusion, this case highlights the need to update the current standard for the diagnosis of BBS and to clarify the role of genetic testing: due to evolution of the phenotype and the increasing availability of genetic testing, genetic testing may precede clinical diagnosis. A less strict application of the diagnostic model is indicated when identified mutations are classified as ‘likely pathogenic’ or ‘pathogenic’, ultimately leading to earlier diagnosis and thus intervention.

## Declaration of interest

The authors declare that they have no competing interests that might be perceived as prejudicing the impartiality of the case study reported.

## Funding

This study did not receive any specific grant from any funding agency in the public, commercial, or not-for-profit sectors.

## Patient consent

Written informed consent was obtained from the patients for the publication of this article and the accompanying images.

## Author contribution statement

All authors participated in the care of the patients and their family. NVR and DH performed data collection and interpretation. NVR drafted the manuscript. All authors read and approved the final version of the manuscript.
